# Immune Response After Vaccination against SARS-CoV-2 in the Elderly in the Czech Republic: Hana Tomaskova

**DOI:** 10.1093/eurpub/ckac130.228

**Published:** 2022-10-25

**Authors:** H Tomaskova, J Martinek, J Janosek, H Zelena, A Kloudova, E Jezo, V Kral, H Sturcova, J Pohorska, R Madar

**Affiliations:** 1 Department of Epidemiology and Public Health, University of Ostrava, Ostrava, Czechia; 2 Institute of Public Health Ostrava, Ostrava, Czechia; 3 Institute of Public Health Ústí nad Labem, Ústí nad Labem, Czechia; 4 4Centre for Health Research, Faculty of Medicine, University of Ostrava, Ostrava, Czechia

## Abstract

**Background and Aims:**

Elderly, especially those with chronic diseases, are a population at high risk of a severe course of the SARS-CoV-2 infection. The study aimed to investigate the complex immune response after vaccination in nursing home residents older than 65 years depending on the previous COVID-19 status and vaccine brand.

**Methods:**

375 participants participated in the study in September-October 2021. IgG antibodies against spike protein and nucleocapsid protein, the titer of virus neutralization antibodies, and cellular immunity (interferon-gamma release assay) were tested in elderly nursing home residents vaccinated with Pfizer, Moderna 30-31 weeks after the completion of vaccination and in those vaccinated with AstraZeneca 23 weeks before sampling. The prevalence with 95% confidence intervals (CI) was evaluated in Stata version 17.

**Results:**

In total, 95.2% (95% CI: 92.5%-97.1%) of samples had positive results of anti-S IgG, 92.8% (95% CI: 89.7%-95.2%) were positive in virus neutralization assay and 89.0% (95% CI: 84.5%-92.5%) in the interferon-gamma-releasing assay (indicator of cellular immunity). Even though the immune response to the Pfizer and Moderna vaccines were evaluated after a longer period than AstraZeneca, the immune response in residents vaccinated with these vaccines were superior. All immune parameters in vaccinated individuals were significantly higher in convalescent residents than in those who had not been infected with COVID. No case of COVID-19 occurred during the vaccination-to-test period.

**Conclusions:**

High immune response was found in elderly nursing homes residents (65 years and older) after 5-7 months after vaccination against SARS-CoV-2. In particular, vaccinated convalescents showed high immune responses. It appears that such residents are much better protected from COVID-19 than those who are only vaccinated.

**Key messages:**

- In a random sample of elderly nursing homes residents, high immune responses were found 5-7 months after the completion of vaccination against SARS-CoV-2 in the Czech Republic.
- During the vaccination-to-test period (5-7 months) no case of COVID-19 occurred in the samples of Elderly Nursing Homes Residents in the Czech Republic.

